# Needle Radiofrequency Combined with Topical Exosome Therapy for Moderate to Severe Acne

**DOI:** 10.3390/life15020141

**Published:** 2025-01-21

**Authors:** Jovian Wan, Song Eun Yoon, Sky Wong, Inneke Jane Hidajat, Henry Tanojo, Atchima Suwanchinda, Kyu-Ho Yi

**Affiliations:** 1 Medical Research Inc., Wonju 06010, Republic of Korea; contact.drjovian@gmail.com; 2 BrandnewAesthetic Surgery Clinic, Seoul, Republic of Korea; 3 Leciel Medical Centre, Hong Kong; drskywong@gmail.com; 4 Department of Dermatology, Faculty of Medicine, Atma Jaya Catholic University of Indonesia, Jakarta 1440, Indonesia; 5 Melania Clinic, Surabaya 60264, Indonesia; 6 Division of Dermatology, Department of Medicine, Faculty of Medicine Ramathibodi Hospital, Mahidol University, Bangkok 73170, Thailand; dr.atchima@gmail.com; 7 Division in Anatomy and Developmental Biology, Department of Oral Biology, Human Identification Research Institute, Yonsei University College of Dentistry, 50-1 Yonsei-ro, Seodaemun-gu, Seoul 03722, Republic of Korea; 8 Maylin Clinic, Apgujeong, Seoul 06015, Republic of Korea

**Keywords:** acne vulgaris, radiofrequency therapy, exosomes, skin regneration, sebaceous glands

## Abstract

Objective: This case series aims to evaluate the efficacy, patient satisfaction, and safety of combined needle radiofrequency (RF) and topical exosome therapy for moderate to severe acne. Methods: This study involved 22 patients (12 females and 10 males, ages 18–35) with moderate to severe acne who underwent combined needle RF and topical exosome (Xomage, Zishel Bio Inc., Seoul, Republic of Korea) treatments. Each patient completed between 6 and 10 sessions, conducted weekly over three-week intervals. Acne severity was assessed using the Investigator’s Global Assessment (IGA) scale, while patient satisfaction was measured on a 5-point Likert scale. Clinical photographs were taken at baseline and after the final treatment session. Results: All patients showed improvement in acne severity with a mean decrease in IGA score of 2.5 points from baseline to the final assessment. Patient satisfaction was high, with the majority expressing satisfaction in skin texture and acne reduction. Conclusion: Needle RF combined with topical exosome therapy appears to be an effective treatment for reducing acne lesions and improving skin quality, demonstrating a strong safety profile and high patient satisfaction.

## 1. Introduction

Acne vulgaris is one of the most common dermatological conditions worldwide, affecting up to 85% of adolescents and many adults. Its pathogenesis is multifactorial, with key contributors including sebaceous gland hyperactivity, follicular hyperkeratinisation, and bacterial proliferation by *Cutibacterium acnes*, which leads to inflammation and the formation of lesions such as comedones, papules, pustules, and nodules. In moderate to severe cases, acne often requires aggressive and sustained interventions to achieve satisfactory outcomes [1]. Traditional pharmacological treatments such as topical retinoids, benzoyl peroxide, antibiotics, and oral isotretinoin are commonly used, but their effectiveness is often limited by side effects, including dryness, irritation, photosensitivity, and the potential for antibiotic resistance [2,3,4]. Furthermore, patient adherence to long-term treatments can be poor due to treatment fatigue and dissatisfaction with a slow or incomplete response, highlighting the need for alternative approaches.

Acne’s presentation is highly heterogeneous, with severity, distribution, and triggers varying significantly among individuals. Hormonal fluctuations, especially increased androgens, contribute to exacerbations, particularly in adult women, with outbreaks often occurring along the jawline and chin. Dietary factors, such as high glycaemic index foods and dairy, have also been linked to worsened acne severity [5]. More recently, the gut–skin axis has been identified as a potential factor in acne pathogenesis, with dysbiosis in gut microbiota possibly propagating systemic inflammation and exacerbating skin conditions [6].

Acne not only has physical consequences but also significantly impacts psychological well-being, often leading to reduced self-esteem, anxiety, and depression. The stigma associated with acne can lead to social withdrawal, which perpetuates emotional distress. Addressing both the clinical and psychological aspects of acne can improve patient satisfaction and treatment adherence [7,8]. With the limitations of pharmacological therapies, device-based treatments have become increasingly important in providing targeted, non-systemic solutions.

The chronic, recurrent nature of acne often leads to frustration with conventional treatments. While topical and systemic therapies remain first-line approaches, their limitations, such as the delayed onset of results and adverse effects, prompt patients to seek alternative options [9,10]. This highlights the need for innovative therapies that offer faster, safer, and more comprehensive outcomes.

In response to these challenges, dermatological devices have emerged as promising alternatives for acne management. Needle radiofrequency (RF) is one such innovation that has gained attention for its ability to deliver controlled thermal energy directly into the dermis [11,12]. This technique targets sebaceous glands, disrupting their activity and ability to reduce sebum production. Moreover, RF’s thermal effects stimulate collagen remodelling, which not only addresses active lesions but also improves post-acne scarring. Unlike traditional laser treatments, RF minimises epidermal damage, thereby reducing the risk of post-inflammatory hyperpigmentation (PIH), particularly in the patients with Fitzpatrick skin types III and IV [13]. This makes it a safer option for a wider range of skin types [9,14].

Exosome therapy, derived from mesenchymal stem cells, is another promising advancement in regenerative medicine. Rich in bioactive molecules such as growth factors, cytokines, and proteins, exosomes facilitate cellular communication, modulate inflammation, and promote tissue repair [10]. These properties have demonstrated promise in accelerating wound healing, improving skin texture, and enhancing the results of both ablative and non-ablative treatments in dermatology. When applied topically, exosomes can support post-treatment recovery and amplify the effects of device-based therapies, making them an innovative approach to aesthetic medicine. Their wide-ranging benefits, from promoting skin regeneration to anti-ageing applications, are solidifying their role in both therapeutic and aesthetic dermatology [15,16]. The combination of needle RF and exosome therapy leverages their complementary mechanisms. RF reduces sebaceous gland activity and inflammation, directly targeting the causes of acne, while exosomes enhance dermal regeneration, supporting both short-term acne reduction and long-term skin healing. This integrated approach reflects the growing trend in dermatology towards holistic, patient-centred care that addresses both the symptoms and the underlying skin conditions.

In addition to their clinical efficacy, these modalities meet the increasing patient preference for treatments that provide rapid results with minimal downtime. This makes needle RF and exosome therapy highly attractive to individuals seeking effective acne management without the prolonged side effects or the risks associated with conventional treatments. As patients increasingly opt for minimally invasive options, the combined use of RF and exosome therapy offers a convenient and effective solution that aligns with the modern expectations of dermatological care.

This case series explores the synergistic effects of combining needle RF with topical exosome therapy for managing moderate to severe acne. The aim is to offer a comprehensive solution that targets both the inflammatory and structural components of acne, providing an effective, patient-centred treatment.

## 2. Methods

### 2.1. Patient Selection and Inclusion Criteria

A total of 22 patients with moderate to severe acne vulgaris were enrolled in this case series. The cohort consisted of 12 females and 10 males, ranging in age from 18 to 35 years (mean age of 27.1 years). Eligibility criteria included an IGA acne severity score of 3 (moderate) to 4 (severe), Fitzpatrick skin types III–IV, and an absence of recent acne treatments such as oral retinoids or antibiotics for at least three months prior to enrolment. Patients with a history of photosensitivity, autoimmune disorders, pregnancy, or recent facial treatments (chemical peels or laser therapies within six weeks) were excluded.

This study was conducted in accordance with the Declaration of Helsinki and approved by the Ethics Committee of the Komisi Etika Penelitian, Fakultas Kedokteran dan Ilmu Kesehatan, Universitas Katolik Indonesia Atma Jaya (protocol code 01/10/KEP-FKIKUAJ/2024, approved on 21 October 2024). Informed consent was obtained from all subjects involved in this study.

### 2.2. Treatment Protocol

Patients received combined needle RF and topical exosome therapy. Each session began with needle RF treatment administered to the entire face, followed immediately by the application of a topical exosome (Xomage, Zishel Bio Inc., Seoul, Republic of Korea). This protocol was conducted at weekly intervals, with each patient receiving a minimum of six and a maximum of ten sessions, based on their initial severity and response to treatment.

### 2.3. Needle Radiofrequency Treatment

The RF procedure involves a minimally invasive microneedle RF system featuring 25 non-insulated bipolar electrodes arranged in 5 × 5 parallel rows. The treatment protocol consists of 5 sessions of microneedle RF. For each session, fractional microneedle RF is applied with the following parameters: RF intensity set at 50%, needle depth of 0.5 mm, pulse duration of 50 ms, and a single pass on the right side of the face. The total energy delivered per treatment is 2.3 joules in pulsed cycles. Topical anaesthetic (lidocaine-prilocaine cream) was applied 30 min before each session to ensure patient comfort.

### 2.4. Topical Exosome Application

After the RF treatment, a concentrated exosome serum derived from adipose-derived mesenchymal stem cells (ADMSCs) was applied to the treated areas. The serum was left on the skin for 10–15 min to maximise absorption, with no additional post-care required. The exosome serum contains 10 billion exosome particles per ampoule (100 mg per vial) and is formulated with ADMSC-derived exosomes suspended in stem cell media. It is enriched with CELLEXIR activator, six growth factors, six peptide complexes, four types of plant-derived extracellular vesicles, as well as panthenol and sodium hyaluronate.

During the 10–15 min application period, the serum was fully absorbed into the skin, and no removal or further treatment was necessary. Patients were instructed to refrain from washing the treated area for six hours to ensure optimal absorption and maximise the therapeutic effects of the serum.

## 3. Outcome Measures

### 3.1. Severity of Acne

Acne severity was evaluated using the Investigator’s Global Assessment (IGA) scale, which rates acne from 0 (clear) to 4 (severe). The IGA scale was assessed at baseline, halfway through the treatment (after three sessions), and at the final assessment point.

### 3.2. Patient Satisfaction

Patient-reported satisfaction was measured on a 5-point Likert scale (one = very dissatisfied, five = very satisfied) at the final assessment visit. The patients rated their satisfaction with the overall acne improvement, skin texture, and reduction in lesion count.

### 3.3. Photographic Documentation

**Figure 1:**
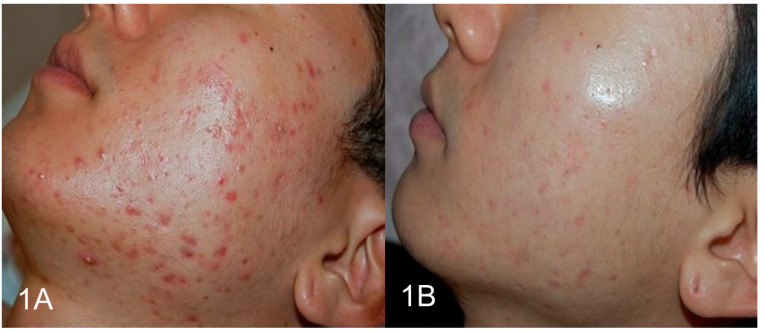
(**A**,**B**) A 25-year-old male patient displayed a significant reduction in inflammatory acne lesions and an improvement in skin texture post-treatment.

**Figure 2:**
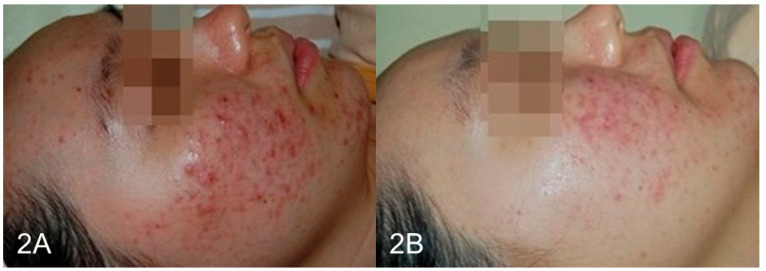
(**A**,**B**) A 27-year-old female patient exhibited considerable acne clearance and a smoothened skin texture after the full treatment series.

Clinical photographs were taken under standardised lighting and camera settings at the baseline, mid-treatment, and post-treatment sessions. Figure 1A,B illustrates the pre- and post-treatment images of a 25-year-old male patient, while Figure 2A,B shows the images for a 27-year-old female patient.

### 3.4. Safety and Tolerability

Side effects were monitored at each session and the follow-up visits, with patients reporting any adverse symptoms such as erythema, oedema, or pain. These symptoms were documented and rated based on severity.

### 3.5. Statistical Analysis

The quantitative data were analysed using descriptive statistics. Paired *t*-tests were used to compare mean changes in the IGA scores and the patient satisfaction scores from baseline to the final assessment, with statistical significance defined as *p* < 0.05.

## 4. Results

### 4.1. Patient Demographics

Of the 22 patients included, 18 completed the full series of ten sessions, while four received six to eight sessions based on their clinical improvements. The average age was 27.1 years, with skin types predominantly being Fitzpatrick III and IV.

### 4.2. Efficacy of Combined Therapy

#### 4.2.1. Improvement in Acne Severity

All the patients exhibited notable improvements in acne severity, with IGA scores decreasing by an average of 2.5 points from baseline to the final assessment (Table 1). At the final assessment, 16 patients achieved an IGA score of one (almost clear), while 6 patients achieved a score of two (mild). Notably, the inflammatory lesions showed greater reductions compared to the non-inflammatory lesions, aligning with the RF’s thermal effects on sebaceous glands.

#### 4.2.2. Patient Satisfaction

The patient satisfaction scores averaged 4.2 on the Likert scale, with 18 patients reporting scores of four (satisfied) and 4 patients reporting scores of five (very satisfied) (Table 1). The patients commonly reported visible improvements in skin texture, a reduced lesion count, and improvements in overall skin appearance.

#### 4.2.3. Safety and Side Effects

The combination therapy was well tolerated, with minimal adverse effects reported across the patient group. All patients experienced mild erythema immediately following the needle RF procedure, which resolved within approximately 45 min after the application of topical exosomes. This quick resolution highlights the soothing and anti-inflammatory properties of exosomes, facilitating a rapid recovery post-treatment. No cases of post-inflammatory hyperpigmentation, scarring, or other long-term side effects were observed, further underscoring the safety profile of needle RF in combination with exosome therapy.

## 5. Discussion

This case series demonstrated that combining needle RF with topical exosome therapy is an effective and well-tolerated treatment for moderate to severe acne. The observed clinical outcomes align with the proposed mechanisms of action of each therapy. Needle RF, by delivering controlled thermal energy into the dermis, targets sebaceous glands and reduces sebum production, a key contributor to acne pathogenesis [13]. This focused approach minimises epidermal damage, which is especially beneficial for patients with Fitzpatrick skin types III and IV who are more prone to PIH with traditional laser treatments. Our findings indicate significant reductions in both the inflammatory and non-inflammatory lesions, along with sustained improvements in IGA scores, further validating the efficacy of RF in addressing the dermal contributors to acne.

A crucial aspect of RF’s success in acne management is its direct effect on sebaceous gland activity. Excess sebum, which provides a substrate for *Cutibacterium acnes* (formerly *Propionibacterium acnes*), is a primary factor in acne lesion development. By modulating sebum production, RF disrupts this cycle, potentially reducing the microbial load and inflammation. This aligns with the current understanding of acne’s pathophysiology, where *Cutibacterium acnes* exacerbate inflammatory responses, particularly in more severe forms of the condition [17,18]. Therefore, RF’s ability to target sebaceous glands not only helps reduce active lesions but also creates a less favourable environment for bacterial proliferation, which is essential for long-term acne management.

Following needle RF treatment, the application of topical exosomes contributes a regenerative component, promoting recovery and supporting the skin’s natural repair processes. Exosomes are known to carry growth factors and signalling molecules that enhance collagen synthesis, reduce inflammation, and support cell-to-cell communication, making them particularly suited to post-RF treatment. In our study, the patients consistently reported improvements not only in acne reduction but also in skin texture and overall skin quality, likely attributable to the regenerative benefits of exosomes. The application of exosomes immediately following an RF treatment may enhance their delivery and absorption into the dermis, maximising their therapeutic impact [19,20].

Exosomes offer more than just anti-inflammatory and regenerative benefits. By modulating key signalling pathways, they help balance the microenvironment of sebaceous glands, reducing inflammation and promoting tissue homeostasis. This dual action complements the mechanical and thermal effects of RF, enhancing its ability to address acne’s underlying causes. Exosomes also facilitate a faster recovery post-RF, minimising downtime, which is a significant advantage for patients seeking effective, convenient treatment.

The synergy between RF and exosomes extends to managing dermal inflammation. RF’s thermal impact on sebaceous glands can temporarily heighten localised inflammation, but the anti-inflammatory cytokines in exosomes help counteract this, accelerating healing. This combined approach not only targets acne’s causative factors but also supports the skin’s post-treatment repair processes.

Patient satisfaction was high, with significant improvements in acne severity and skin texture, as reflected in both subjective feedback and objective measures such as the IGA scores. Compared to traditional treatments, the RF and exosome combination offers quicker results with fewer side effects, making it an appealing option for patients seeking faster improvements and a minimal recovery time. The treatment was well tolerated, with only mild erythema and oedema noted and resolving within 24 h. No cases of hyperpigmentation or scarring were observed, highlighting the safety of this therapy, particularly for darker skin types, who are at a higher risk of post-inflammatory hyperpigmentation with the other treatments. The anti-inflammatory effects of exosomes further enhance safety by mitigating any irritation from RF. The absence of significant adverse effects suggests that this combination therapy is suitable for a broad range of patients.

However, this study is not without limitations. As a single-centre, non-controlled case series, it lacks the comparative rigour of randomised controlled trials. The sample size, while reflective of real-world clinical settings, limits the generalisability of our findings. Furthermore, the follow-up period was restricted to the treatment duration and immediate post-treatment assessments. Future studies could address these limitations by incorporating a larger, more diverse sample, randomising treatment groups, and extending follow-up to better assess the long-term durability of the treatment effects. Comparative studies involving needle RF and exosome therapy versus other acne treatments, such as laser or chemical therapies, would provide more nuanced insights into their relative efficacy and safety.

The absence of a control group further limits our ability to definitively attribute the observed improvements solely to the combined therapy. Future research should include placebo designs to better delineate the specific contributions of RF and exosomes, either as standalone treatments or in combination. Such trials would provide higher-level evidence to validate the promising outcomes seen in this case series. Additionally, further research investigating the histological changes within the dermis following a combined RF and exosome treatment could elucidate the underlying mechanisms driving the observed improvements. An analysis of sebaceous gland histology and inflammatory markers would clarify the precise biological impact of RF, while examining the collagen and elastin changes would deepen our understanding of exosome-related skin remodelling. Such investigations could confirm the anecdotal and clinical observations made in this case series, providing a foundation for broader clinical application and potential integration into standard acne treatment protocols.

In summary, this case series suggests that needle RF combined with topical exosome therapy is an effective and safe treatment for moderate to severe acne, offering dual benefits of acne reduction and skin quality enhancement with minimal downtime and high patient satisfaction. The favourable outcomes support this combination as a viable, non-pharmacological option for patients seeking comprehensive acne management. With further validation through controlled studies, needle RF with exosomes could become a key addition to the armamentarium for acne treatment, particularly for patients seeking alternatives to conventional therapies.

## Figures and Tables

**Table 1:** Investigator’s Global Assessment Scores and Patient Satisfaction on a Five-Point Likert Scale before and after treatment.

Patient ID	IGA Score (Before)	IGA Score (After)	Patient Satisfaction (Likert Scale)
1	4	1	5
2	3	2	4
3	4	1	4
4	3	2	5
5	4	1	4
6	4	1	5
7	4	2	4
8	3	2	5
9	4	1	5
10	3	2	4
11	3	2	4
12	4	1	5
13	3	2	5
14	4	1	4
15	4	1	5
16	4	2	4
17	3	1	5
18	4	1	5
19	3	2	4
20	4	1	5
21	3	2	4
22	4	1	5

## Data Availability

Data are contained within the article.
